# Enhancing the Separation Performance of Chitosan Membranes Through the Blending with Deep Eutectic Solvents for the Pervaporation of Polar/Non-Polar Organic Mixtures

**DOI:** 10.3390/membranes14110237

**Published:** 2024-11-11

**Authors:** Francesco Galiano, Asma Msahel, Francesca Russo, Natalia Rovella, Alfonso Policicchio, Sofiane Ben Hamouda, Amor Hafiane, Roberto Castro-Muñoz, Alberto Figoli

**Affiliations:** 1 Institute on Membrane Technology (CNR-ITM), Via P. Bucci 17/c, 87036 Rende, CS, Italy; f.russo@itm.cnr.it (F.R.); n.rovella@itm.cnr.it (N.R.); a.figoli@itm.cnr.it (A.F.); 2 Laboratory of Water Membrane and Environmental Biotechnology (LMBE), CERTE BP 273, Soliman 8020, Tunisia; asmamsehel@gmail.com (A.M.); amor.hafiane@certe.rnrt.tn (A.H.); 3 Department of Biology, Ecology and Earth Sciences (DiBEST), University of Calabria, Via P. Bucci 12/b, 87036 Rende, CS, Italy; 4 Department of Physics, Università della Calabria, Via P. Bucci, Cubo 31C, 87036 Rende, CS, Italy; alfonso.policicchio@fis.unical.it; 5 Centre for Research on Microelectronics and Nanotechnology (CRMN) of Sousse, Sousse 4054, Tunisia; sofianebenhamouda819@gmail.com; 6 Faculty of Civil and Environmental Engineering, Department of Sanitary Engineering, Gdansk University of Technology, 11/12 Narutowicza St., 80-233 Gdansk, Poland

**Keywords:** deep eutectic solvents, membranes, pervaporation, chitosan, organic/organic separation

## Abstract

This study explores the development of chitosan-based membranes blended with three distinct deep eutectic solvents (DESs) for the pervaporation separation of methanol and methyl *tert*-butyl ether. DESs were selected for their eco-friendly properties and their potential to enhance membrane performance. The chitosan (CS) membranes, both crosslinked and non-crosslinked, were characterized in terms of morphology, chemical composition, wettability, mechanical resistance, and solvent uptake. Pervaporation tests revealed that incorporating DESs significantly enhanced the membranes’ selective permeability toward methanol, with up to a threefold increase in separation efficiency compared to pristine CS membranes. The membranes demonstrated a strong dependence on feed temperature, with higher temperatures improving permeation flux but reducing separation factor. Crosslinking with glutaraldehyde further increased membrane selectivity by reducing free volume into the polymer matrix. These findings underscore the potential of DESs as green additives for improving the performance of biopolymer membranes, making them promising candidates for efficient and eco-friendly organic–organic separations.

## 1. Introduction

The preparation of polymeric membranes using more sustainable and environmentally friendly solvents is a highly relevant topic, as evidenced by the growing number of publications in this area. A series of green solvents, in fact, have been already proposed and employed with satisfactorily results for the preparation of membranes in different geometries and exhibiting different morphologies [1]. Among them, ionic liquids (ILs) and deep eutectic solvents (DESs) are considered as a new generation of sustainable solvents that have been recently explored for the preparation of membranes [2,3]. DESs and ILs share some comparable physicochemical properties, such as the low vapor pressure, the non-flammability, the high chemical stability and, in some cases, the solubility in water [4,5]. In particular, DESs can be typically synthesized from green precursors without the use of any additional organic solvent by connecting a hydrogen-bond acceptor (HBA) and a hydrogen-bond donor (HBD) through hydrogen-bond formation [6,7]. At the same time, DESs are widely appreciated for their biocompatibility, exceptional biodegradability, reusability, low preparation cost, and low melting point [8,9]. For these reasons, they have been proposed for several applications, such as organic reactions [10,11,12], CO_2_ adsorption [13], metal electrodeposition [14], liquid–liquid extraction [15] and biotransformations [16]. In the field of membrane technology, DESs have been recently used both as polymer solvents [17] and as liquid additives immobilized within the membrane matrix to enhance its separation properties [8,18].

Very recently, a DES made of zinc chloride and acetamide was used, for instance, by Pulyalina et al. [19] as a modifier of membranes based on polyamide-imide (Torlon) applied in pervaporation (PV). The incorporation of the DES resulted in membranes with a different morphology (the formation of spherical domains at high DES concentrations) and improved hydrophilicity. When tested in PV, for a water–isopropanol separation, DES-based membranes were more permeable to water with respect to pristine Torlon membranes but less selective as a consequence of a more porous architecture. In another work, Jiang et al. [20] introduced a series of imidazole-based DESs as functional pore-forming additives into polyethersulfone (PES) membranes. This resulted in the formation of more porous membranes characterized by a macrovoid type structure in the support layer and a larger pore size at the membrane surface. Membranes prepared with DESs demonstrated improved water permeability while maintaining excellent separation performance, with the lowest rejection rate for bovine serum albumin reaching up to 97.7%.

Over the last decades, chitosan (CS) has been demonstrated to be a great platform to fabricate high-performance PV membranes due to its highly hydrophilicity. To date, several concepts of CS-based membranes have been developed using different membrane fabrication strategies, including polymer blending, composites, and mixed-matrix membranes, as recently reviewed in detail [21,22]. Interestingly, CS-based membranes have shown extraordinary capability in separating different solvent mixtures via PV technology, such as water–organic, organic water, organic–organic, and seawater desalination [23,24]. In this regard, Castro-Muñoz et al. [8] prepared novel CS membranes using the DES L-proline–sulfolane (molar ratio 1:2) as an additive, and applied the developed membranes in PV for the methanol (MeOH)-methyl *tert*-butyl ether (MTBE) azeotropic separation. From the results, it was found that the addition of DES was able to improve the overall performance of the membranes, which showed a threefold higher separation efficiency respect to the pristine CS membranes. This was likely due to the fact that the chosen DES facilitated the formation of H-bonding interactions, thereby promoting the permeation of MeOH (a more polar molecule) through the membrane. On the basis of these encouraging results, the aim of this work was to carry out a more systematic study by exploring the incorporation of three different non-ionic DESs, exhibiting different chemical–physical properties, to be used as functional additives into CS membranes for the pervaporative separation of the organic–organic MeOH/MTBE azeotropic solution. Specific DESs, such as proline–glucose, 2-pyrrolidone-5-carboxylic acid–sulfolane, and proline–xylitol, were selected based on their composition of naturally derived compounds, low toxicity, cheapness and compatibility with polymer matrices. Their combination with a biopolymer like CS aligns with the goal of making the membrane fabrication process more sustainable.

The produced membranes were characterized in terms of morphology, chemical composition, wettability, mechanical resistance, and solvent uptake. The PV tests were carried out by studying the effect of different feed solution temperatures on membrane flux and separation factor. The crosslinking influence on membrane properties and performance was also evaluated.

## 2. Materials and Methods

### 2.1. Materials

MeOH was purchased from VWR chemicals (Milan, Italy) and MTBE 99.9% was obtained from Sigma Aldrich (now Merck, Milan, Italy). Chitosan, specified as medium molecular weight, was bought from Sigma Aldrich. The precursors of the synthesized DESs were acquired as follows: sulfolane (purity ≥ 99%, Alfa Aesar); 2-pyrrolidone-5-carboxylic acid (purity ≥ 99%, Sigma Aldrich); sulfuric acid (ACS purity, Avantor Performance Materials Poland S.A.); L-proline (purity ≥ 98%, Sigma Aldrich, Poznan, Poland); xylitol (pure, WarChem, Warsaw, Poland); glucose (pure, WarChem). Hydrochloric acid (analytical reagent) was purchased from POCH S.A. (Gliwice, Poland) while glutaraldehyde (25% solution) and acetic acid (purity ≥ 99%) were purchased from Sigma Aldrich (now Merck, Milan, Italy).

### 2.2. Membrane Preparation

As for DES synthesis, proline–glucose (PRO:GLU), 2-pyrrolidone-5-carboxylic acid–sulfolane (PCA:SULF) and proline–xylitol (PRO:XYL) solvents were prepared following the same procedures already documented by our research group [25,26,27]. Table 1 enlists the chemical structure of the DES precursors, molar ratios, and codes used over this study. Once the DESs were prepared, they were independently used in the preparation of CS membranes. In dense membrane preparation, the so-called dense-film casting method was applied to fabricate the flat membranes. Basically, CS dope mixtures (1.5 wt%) were prepared using an aqueous solution containing acetic acid (2 wt%), which was continuously stirred overnight after preparation. Thereafter, each DES (5 wt%), which represents the optimal DES percentage at a given molar ratio in respect to CS, was added into the dope polymer solution. The right DES percentage was preliminarily studied and determined in previous research [26]. The CS/DES dissolution was mixed for 4 h, followed by the chemical in situ crosslinking when required. Then, glutaraldehyde (100 µL), followed by HCl (100 µL), were doped to the final CS/DES dissolution. Given 15 min mixing, the dope mixtures were independently cast onto Petri plastic dishes for film formation. The flat membranes were exposed to solvent evaporation over 48 h. The membrane specimens presented ca. 30 μm thickness, determined by microscopy. The membranes prepared are referred to throughout the manuscript by the codes “CS” or “xCS” (for crosslinked membranes), followed by the code of the DES incorporated into their matrix.

### 2.3. Membrane Characterization

#### 2.3.1. FT-IR and Scanning Electron Microscopy (SEM)

FT-IR was measured in membrane specimens by means of a Nicolet iS10 spectrometer (from Thermo Fisher Scientific, Rodano, Italy), equipped with deuterated triglycine sulfate (DTGS) detector and a Golden Gate diamond ATR. The spectra data were taken in the range 4000–400 cm^−1^, with a resolution of 16 cm^−1^.

The morphology of the membranes was evaluated by means of a SEM instrument (Zeiss EVO, MA100, Assing, Monterotondo, Italy). Membrane samples were sputter-coated before the analyses with a thin layer of gold using a Quorum Q150 RS sputter machine (Quorum Technologies, Lewes, UK) (4 min was set as the duration time of the cycle).

#### 2.3.2. Contact Angle and Mechanical Tests

Contact-angle values were measured using ultra-pure water (5 μL drop) by means of a CAM 200 contact-angle instrument (CAM200, KSV Instruments, Espoo, Finland). The mechanical properties were determined at room temperature by means of a Zwick/Roell Z2.5 test unit (BTC-FR2.5TN-D09, Zwick Roell Group, Ulm, Germany). Membrane strips were extended at the constant elongation rate of 5 mm min^−1^ until they broke, and the elongation at break and Young’s Modulus were, then, determined.

#### 2.3.3. Uptake Experiments

The solvent uptake (swelling degree) of all investigated membranes was evaluated for MeOH:MTBE at the azeotropic point (14.3% MeOH and 85.7% MTBE). For each membrane, three different pieces were weighed (W_d_) with a digital balance (Gibertini, Crystal 500, Novate Milanese, Italy) and immersed in the swelling solution to reach the equilibrium for 24 h at room temperature. After this time, the wet membrane pieces were removed, wiped with tissue paper to remove excess liquid from the surface, and weighed again (W_s_). The degree of swelling (DS) was calculated as follows (Equation (1)):

(1) $$DS(\%)=\frac{W_{s}-W_{d}}{W_{d}}\times 100$$

W_s_ and W_d_ correspond to the wet and dry membrane weights, respectively.

### 2.4. Pervaporation Tests

PV tests were performed using the setup schematically shown and described elsewhere [1]. Basically, a 300 mL jacketed reservoir was filled with an azeotropic MeOH/MTBE (14.3%/85.7%) mixture in contact with the membrane with an active area of 9.6 cm^2^. The feed solution was maintained at a specific temperature (25, 35, or 45 °C) using a digital circulating bath (Thermo Electro Corporation, HAAKE P5, Thermo Fisher Scientific, Rodano, Italy). The vacuum on the permeate side was maintained at 0.04 mbar by means of a vacuum pump (Edwards XDS 5, Cinquepascal, Trezzano sul Naviglio Milano, Italy). During the 5 h experiment, the vapor permeate was condensed and collected in a cold trap placed in a liquid nitrogen and analyzed at 25 °C using an Abbe 60 refractometer (60/DR, Bellingham + Stanley Ltd., Nottingham, UK). After determining the permeate composition, the total flux (J) (Equation (2)), the partial flux (J_i_) for each component *i* (Equation (3)), and the separation factor (α) (Equation (4)) were calculated according to the following equations:

(2) $$\;J=\frac{Q}{At}$$

where Q is the weight of the permeate in kg, A corresponds to the active membrane area (m^2^), and t is the operating time (expressed in h).

(3) $$J_{i}=w\cdot J$$

where J_i_ represents the fraction of permeate component *i* in the total permeate flux (J) and P_i_ corresponds to its weight fraction.

(4) α=YMeOHYMTBExMeOHxMTBE

where y and x represent the weight fraction of the two components in the permeate and feed, respectively.

The dependence of permeate flux on the temperature was determined by applying the Arrhenius model, as reported in Equation (5).

(5) $$J=J_{0}\cdot exp\left(-\frac{E_{p}}{R\;T}\right)$$

where *J*_0_ and *E*_p_ are the pre-exponential factor and the apparent activation energy, respectively; *R* is the gas constant, and *T* is the absolute temperature.

### 2.5. Hansen Solubility Parameters (HSPs)

HSPs are physicochemical parameters made up of three components, each corresponding to different types of molecular interactions that occur between materials:

- δ_d_: dispersive interactions (van der Waals forces)
- δ_p_: polar interactions
- δ_h_: hydrogen bonding

The HSPs of DESs were calculated using the method of Hoftyzer Van Krevelen [17,28,29,30] through the following Equations (6)–(8) for each component of the molecule:

(6) $$\delta_{d}=\frac{\sum Fdi}{V}$$

(7) $$\delta_{p}\;=\frac{\sum Fpi}{V}$$

(8) $$\delta_{h}\;=\sqrt{\frac{\sum Fhi}{V}}$$

where Fdi, Fpi and Fhi are the contributions of each molecular component for dispersion, polar, and hydrogen-bonding forces, respectively, and v is the molar volume.

The HSPs for the whole molecule of DESs and NADESs were calculated by using Equation (9) according to Rasool et al. [31], Li et al. [32], and Boston [33]:

δ_x_ = (S1·X% + S2·X%)/100 (9)

where δ_x_ is the HSP (δ_d_, δ_p_ or δ_h_) of the entire DES molecule, S1 and S2 represent the HSP (δ_d_, δ_p_ or δ_h_) of the single solvent components (HBD and HBA), and X is the percentage of their molar ratio.

## 3. Results and Discussion

### 3.1. Characterization of Produced Membranes

Figures S1–S8 in the Supplementary Materials show the FT-IR spectra of the membranes produced with and without DES. The IR spectrum of the pristine CS and xCS membranes (Figures S1 and S2) showed the characteristic peaks of the CS molecule.

A band in the region 3262 cm^−1^ corresponds to N-H and O-H stretching, as well as the intramolecular hydrogen bonds in the amino group of the chitosan molecules. The absorption bands at around 2884 cm^−1^ could be correlated to C-H stretching. The bands at around 1628 cm^−1^ (C=O stretching of amide I) and 1310 cm^−1^ (C-N stretching of amide III) suggest residual N-acetyl groups [8].

The CH_3_ symmetrical deformations were confirmed by the bands at around 1377 cm^−1^. The absorption band at 1151 cm^−1^ indicate asymmetric stretching of the C-O-C bridge. Finally, the bands at 1065 and 1025 cm^−1^ correspond to C-O stretching [34].

For the membranes produced with the DES containing proline (CS PRO:GLU and CS PRO:XYL) (Figures S3 and S7), it is possible to observe an overlap of the spectra in the range of 3000–2900 cm^−1^, which are ascribed to O–H, N–H and C–O oscillations in chemical functionalities of the eutectic mixture and polymer (i.e., polymer and DES), as already observed by Jakubowska et al. and Castro-Muñoz et al., who fabricated CS/DES membranes [26,35]. For the membrane CS PRO:XYL, the broader peak observed at 3000 and 3400 cm^−1^ could be due to the contribution of the–OH groups of the xylitol molecule [36]. For the membranes prepared with CS PCA:SULF (Figure S5), it is possible to observe a broadening of the peak in the region of 1633–1531 cm^−1^ as a possible shift in the carbonyl C=O stretch of the carboxylic acid.

**Figure 1:**
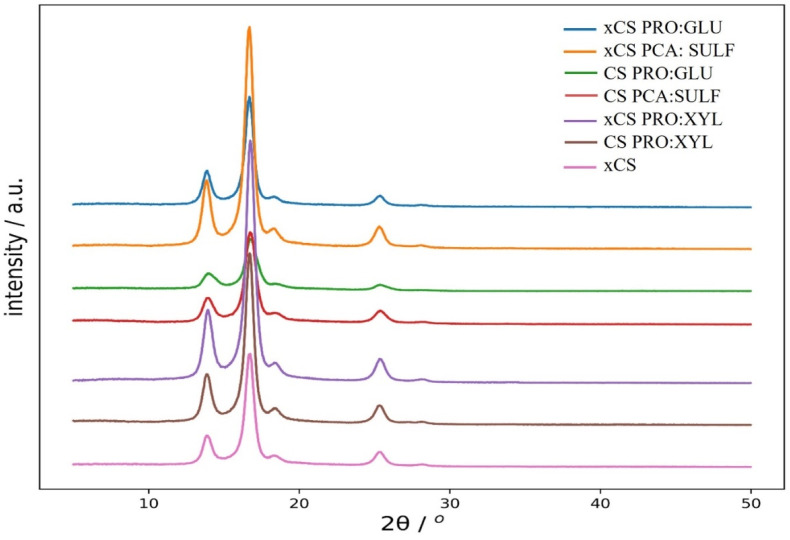
XRD spectra of investigated membranes.

The crystal structure of CS membranes with and without DES is reported in Figure 1. XRD is useful, in the case of composite materials, to study the interactions among the different components. The pristine xCS membrane showed two main strong characteristic diffraction peaks at about 2θ = 14° and 17° [37], indicating a high crystallinity of the polymer. The presence of hydroxyl and amino groups are responsible for the strong intermolecular and intramolecular hydrogen bonds which, along with the regular structure of CS, provide a high degree of crystallinity [38,39].

The characteristic peaks of xCS appeared at the same position after doping with DES, independently of their different chemical structure and the effect of crosslinking. This is an indication of the uniform distribution of the DES into the CS membranes with a preservation of the polymer semi-crystalline structure [40].

By looking at Figure 1, there is a significant variation (intensification of peak patterns) in the diffraction pattern of the DES-doped CS membranes compared with bare xCS. In particular, three DES-doped membranes (xCS PRO:XYL, CS PRO:XYL, xCS PCA:SULF) showed an increase in the peak intensities, suggesting that the crystallinity of CS membranes has been influenced by their addition. This could be explained by the fact that the incorporation of these DESs may, in certain cases, enhance intermolecular bonding between chitosan molecules, making the chains more rigid and restricting their movement. This, in turn, affects the crystallinity of the membranes, leading to an improvement in their elastic properties, such as elongation at break (see Figure 1). The addition of PRO:GLU was the only case where a significant lower peak intensity, with respect to xCS, was observed. In the cases of xCS, PRO:GLU, and CS PCA:SULF, no decrease in peak intensity and, therefore, in crystallinity was observed.

**Figure 2:**
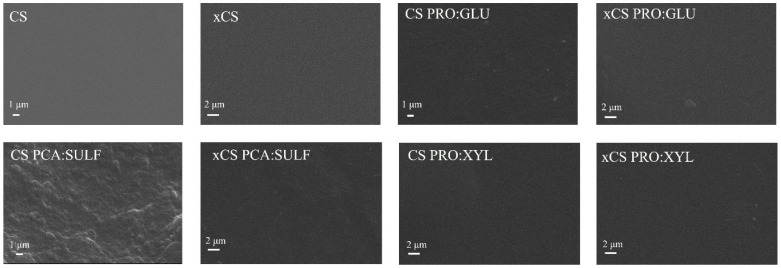
SEM images of the top surfaces of investigated membranes (for a fair comparison, all the images were taken at the same magnification of 10,000×).

The surface morphologies of the prepared membranes are shown in Figure 2. All the membranes displayed a dense and compact surface, typical of membranes prepared by a solvent evaporation technique. The addition of DES into the CS matrix did not cause evident phase separation or an alteration of the surface morphology, which resulted in defect-free and uniform structures regardless of the DES employed. This result can be considered an indication of the proper dispersion and incorporation of the employed DES into CS membranes.

**Figure 3:**
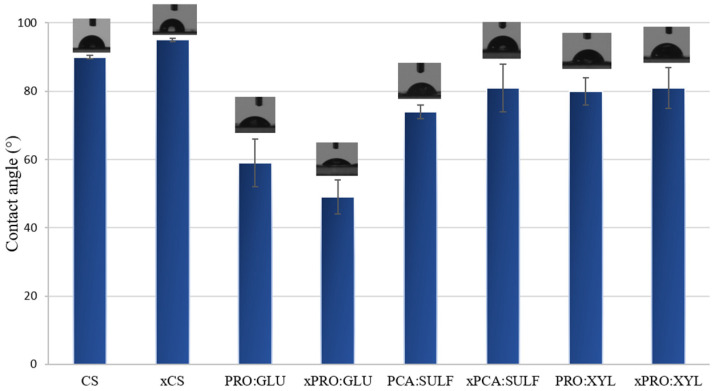
Contact angle values of investigated membranes.

The results of the contact-angle measurements of the prepared membranes are presented in Figure 3. In particular, CS and xCS membranes presented contact values of 90° and 95°, respectively. The contact-angle values of CS-based membranes, reported in the literature, range from 74° to 90° [41,42] and are influenced by many factors such as the degree of acetylation, which generally foster the hydrophilicity of the membrane [43]. On the contrary, the DES-based membranes displayed lower contact-angle values and, hence, a superior hydrophilic moiety. In particular, the membranes prepared with the DES PRO:GLU presented the lowest contact angle (between 49 and 59°). Despite the hydrophobic nature of proline, glucose is in fact a polar molecule, bearing different hydroxyl groups able to attract polar molecules (like water and MeOH) to establish hydrogen bonds. For the membranes prepared with DES PRO:XYL, xylitol is a natural carbohydrate classified as a sugar alcohol with five hydroxyl groups in its structure. Despite its hydrophilic nature, its partition coefficients, with respect to glucose, are higher, indicating a relatively lower hydrophilic character [44]. This could explain the higher contact-angle values (about 80°) of the membranes prepared with this DES. Finally, the membranes prepared with the PCA:SULF showed a contact angle between 74 and 81°. Compared to our previous analysis [25], this latter membrane composition seems to be more hydrophilic; however, water contact-angle measurement, to some extent, provides insight into the membrane surface geometry, i.e., surface roughness, but does not imply any correlation between the intrinsic properties and the chemical nature of the composite structure [45]. In other words, a hydrophilic polymer can be tuned to possess a more hydrophobic geometry by changing the surface roughness.

**Figure 4:**
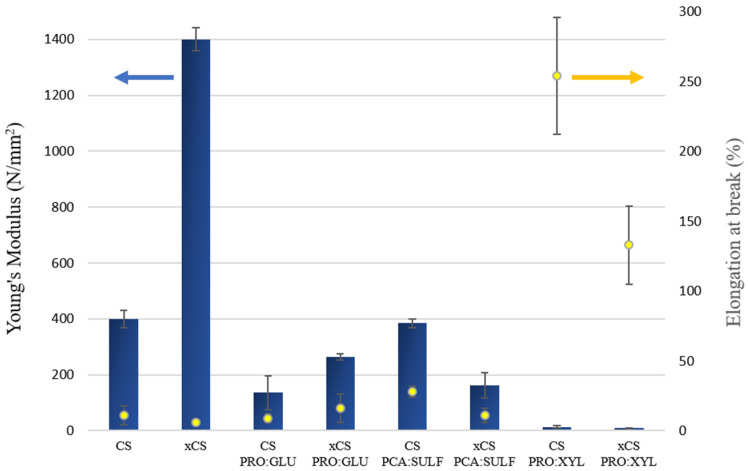
Mechanical properties of investigated membranes: Young’s Modulus (blue bars) and elongation at break (yellow dots).

Figure 4 shows the mechanical properties in terms of Young’s Modulus and elongation at break of the produced membranes. The reference CS samples exhibited a Young’s Modulus up to 1400 N/mm^2^, as an indication of the stiff properties of the CS membranes (in particular for the crosslinked one), while the elasticity was very low and between 6 and 11%. The incorporation of DES into CS membranes greatly decreased the stiffness of the membranes, which was particularly evident for the membranes prepared with PRO:XYL where the Young’s Modulus dropped up to 9 N/mm^2^. The same behavior has already been observed in our previous work, where a different DES (L-proline–sulfolane) was embedded into a CS matrix [25]. This effect could be related to the fact that the presence of DES can alter the intermolecular interactions among the CS molecules, leading to a plasticization effect [26]. The addition of plasticizers into CS, in fact, is responsible for a transition from a rigid to a softer material with more elastic properties [46]. This aspect can, on one hand, justify the decrease in Young’s Modulus in DES-based membranes and, on the other hand, their increase in elongation at break, especially in the PRO:XYL DES. The same trend was also observed by Wang et al. [47], who incorporated the choline chloride/urea DES into cellulose based membranes. Even in this case, the addition of DES led to a decrease in tensile stress and Young’s Modulus of the resulting membranes, but with an improvement of their elongations, indicating a significant plasticizing effect. The increase in membrane elasticity could be the result of an easier movement possibility of CS chains related to the formation of hydrogen intermolecular bonds between plasticizer and CS [48].

### 3.2. Pervaporation Results

**Figure 5:**
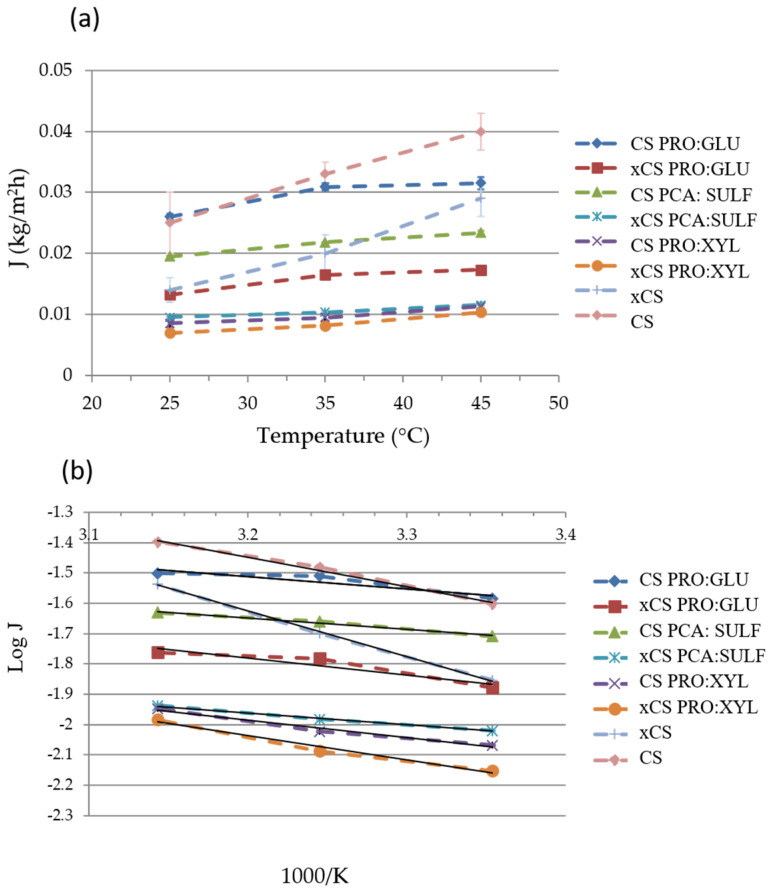
(**a**) The total flux of investigated membranes as a function of feed temperature; (**b**) Arrhenius plot of total flux through the membranes.

Figure 5a displays the PV performances in terms of total flux (J) as a function of the feed temperatures for all the investigated membranes.

**Figure 6:**
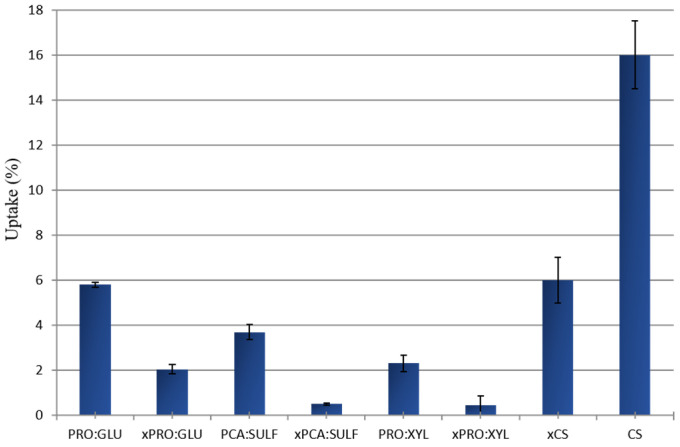
The solvent uptake of investigated membranes at the MeOH/MTBE azeotropic concentration.

As expected, as the feed temperature was increased from 25 to 45 °C, the total permeate flux also increased. This trend could be related to the increase in thermal motion of the polymer chains at higher temperatures, which leads to the creation of more free volumes inside the membrane, fostering the passage of permeating molecules [49]. The plot of total flux as a function of the reciprocal of the absolute temperature shows a linear relationship, indicating an Arrhenius dependence of total flux and operating temperature (Figure 5b). The apparent activation energies (E_a_), calculated from the slopes of the curves and by Equation (5), and shown in Table 2, show positive values very similar to these (except for the pristine CS membranes), indicating that the permeation flux increases with the temperature. For all the investigated membranes, the crosslinking with GA led to a decrease in the total flux, mainly ascribable to a free volume reduction [50]. The CS pristine membrane and the CS PRO:GLU membrane presented the highest total flux values (between 0.025 and 0.04 kg m^−2^ h^−1^), in comparison to all other investigated membranes. The flux trend could be related to the swelling measurement, as shown in Figure 6. These two membranes, in fact, were characterized by the highest swelling degree as an index of their propension to adsorb the permeating molecules present in the feed mixture. On the other hand, the lowest total flux value was found for the xCS PRO:XYL membrane (between 0.007 and 0.01 kg m^−2^ h^−1^), which could be related, also in this case, to its lowest swelling degree at the azeotropic point respect to the other membranes.

**Figure 7:**
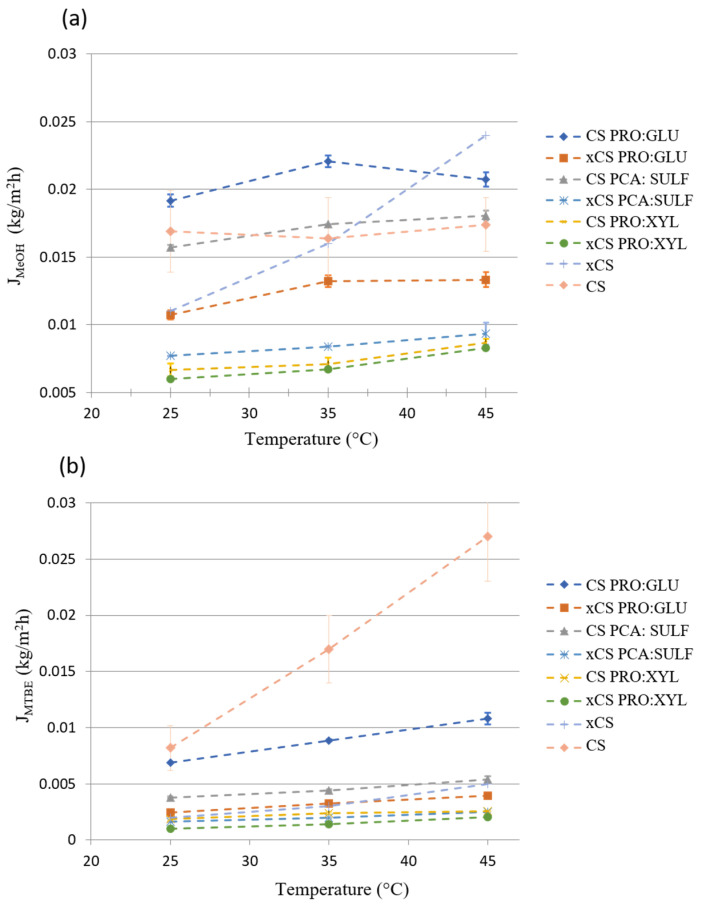
MeOH (J_MeOH_) (**a**) and MTBE (J_MTBE_) (**b**) partial fluxes through the investigated membranes as a function of the feed temperature.

Figure 7a,b display the partial fluxes of MeOH and MTBE. Except for the CS membrane, the MeOH partial fluxes were always higher than the MTBE ones. The partial fluxes, both for MeOH and MTBE, increased as the feed temperature was increased. By looking at the values of E_a_ reported in Table 2, it is possible to observe that, in almost all cases, the values for MTBE were higher than the ones for MeOH, indicating that MTBE flux is more influenced by the temperature than MeOH. This could be ascribed to an enlargement of the polymer free volume with temperature, which promotes the passage of larger molecules such as MTBE [51,52].

According to the calculation of total apparent activation energy, the solvent molecules require more activation energy to be able to permeate across the membrane interfaces when presenting in situ crosslinking. This is in agreement with the fact that crosslinking chemically rearranges the polymer chains, restricting their motion and thus providing higher selectivity. Moreover, the presence of DES clearly reduces the energy required for molecules to permeate the membranes, lowering the E_a_ toward both solvents.

**Figure 8:**
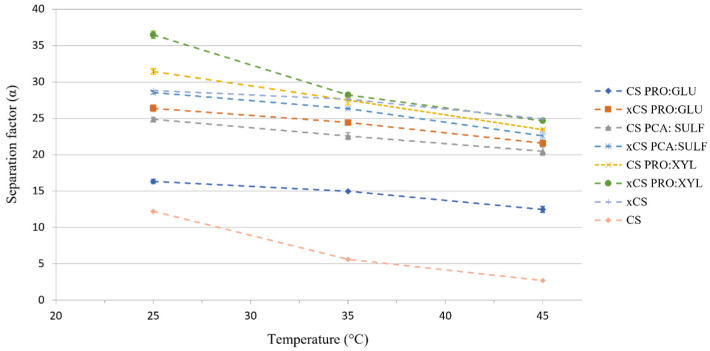
The separation factor of investigated membranes as a function of feed temperature.

In Figure 8, the separation factor of the prepared membranes is shown. The membranes were all methanol-selective according to the separation factor values, and this agrees with the calculated methanol activation energy values reported in Table 2. Methanol, in fact, requires less energy than MTBE to permeate across all formulated membranes, and crosslinking-free membranes presented even lower values of activation energy. Moreover, MeOH molecules have a smaller kinetic molecular diameter (about 3.6 Å) with respect to MTBE (about 6.2 Å), and they are more likely to permeate through membranes’ free volumes. Another aspect which makes the membranes more selective for MeOH is represented by their hydrophilicity. All the membranes, in fact, and, in particular, the ones loaded with DES, displayed a contact angle lower than 90° (see Figure 3) as an indication of their hydrophilic nature, which provides them with a higher affinity for polar molecules (like MeOH).

For all the membranes, the separation factor decreases when the temperature is increased. In line with the free volume theory, the temperature increase is accompanied with an increase in the thermal motion of the polymer chains in the amorphous regions, which results in the formation of wider free volumes more permeable to bigger molecules like MTBE [53]. The same effect explains the higher separation factor, which can be observed for the crosslinked membranes with respect to the non-crosslinked ones. The xCS PRO:XYL membrane was the more selective one, reaching a separation factor of 36.4 (at 25 °C), followed by the CS PRO:XYL membrane (α = 31.4 at 25 °C) and the xCS membrane (α = 28.8 at 25 °C). Pristine CS and CS PRO:GLU membranes were the less selective ones, in line with their higher total fluxes.

The PV results align with the solvent uptake measurements. As illustrated in Figure 6, the xCS PRO:XYL membrane exhibited one of the lowest solvent uptake values, while the less selective membranes (CS and CS PRO:GLU) showed some of the highest. Higher solvent uptake leads to greater membrane swelling, which increases free volume, resulting in higher flux but reduced selectivity.

**Figure 9:**
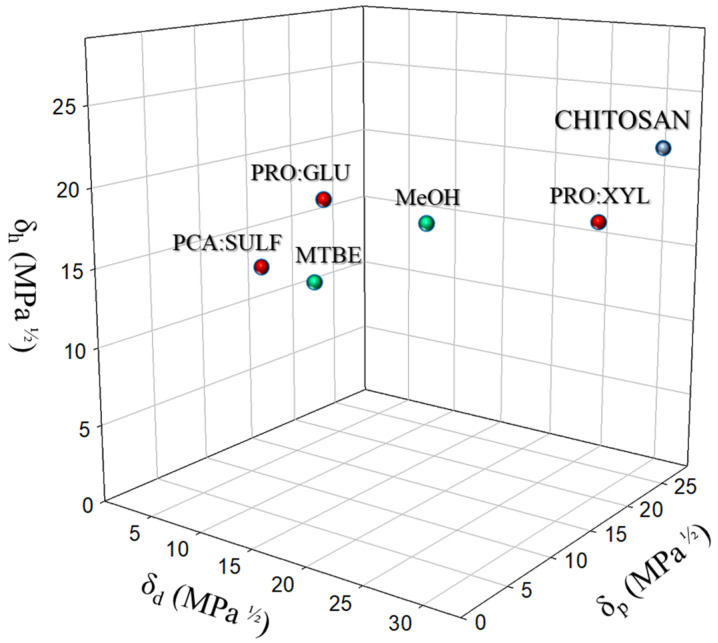
A three-dimensional representation of HSPs including CS, DESs, and solvents used in the pervaporation experiments (MeOH and MTBE).

The compatibility of a polymer with a solvent can be analyzed using the Hansen solubility theory, following the principle of “like dissolves like”. According to the HSPs, polymers are soluble only in solvents with similar HSP values, considering dispersive (δd), polar (δp), and hydrogen-bonding (δh) forces. The HSP values of polymers and DESs were plotted on a three-dimensional space (Figure 9) in order to better visualize the affinity of the investigated DESs for the polymer and the target permeating species (MeOH and MTBE). The closer the HSPs of the polymer are to those of the solvent, the greater the expected solubility. As can be seen from Figure 9, CS exhibits high polar (δp) and hydrogen-bonding (δh) values, suggesting the possibility of strong interactions with polar solvents such as MeOH, which, in fact, is due to its proximity. Conversely, MTBE, being less polar than MeOH, lies further from the CS position, indicating weaker interactions with the chitosan matrix. This distance supports the experimental findings where MeOH is preferentially permeated over MTBE. Based on the positions of the various DESs evaluated, PRO:XYL and PRO:GLU are the closest to MeOH, indicating a strong affinity with the alcohol. However, PRO:GLU is also positioned near MTBE. This may explain the higher selectivity observed in the xCS PRO:XYL membrane, as PRO:XYL is close to MeOH and the furthest from MTBE. Additionally, the closer proximity of this DES to CS suggests a greater compatibility with the polymer, potentially leading to a denser and thus more selective membrane structure. The hypothesis is that PRO:XYL can effectively fine-tune the interaction between the membrane and MeOH, improving its selective separation.

### 3.3. Comparison with the Current State of the Art of PV Membranes Applied for MeOH/MTBE

As reported in Table 3, the comparison of the different concepts of polymer and composite membranes applied for MeOH/MTBE separation is presented. It is worth mentioning that the PV performance of a membrane is affected by important factors, including the membrane characteristics (structure, free volume, nature, etc.), and operating conditions (temperature, pressure, feed concentration, etc.) directly related to the process itself [54]. Therefore, the performance comparison of different PV membranes needs to be carried out in similar operating conditions. Herein, we report the available studies carried out with similar operating parameters. For instance, xCS PRO:XYL seems to exhibit similar selective properties (a separation factor of 36.4) compared to other CS membranes blended with DES, but superior separation factor compared to pristine polymer (PEEKWC, PVA, PLA, CS) and mixed-matrix membranes (such as GO-polyimide). Impressively, polyarylethersulfone with cardo filled with [Cu_2_(bdc)_2_(bpy)]_n_ was reported as outperforming selectivity with values as high as 2300. As for the permeate fluxes, most of the membranes presented very low permeation rates, which are usually obtained in organophilic separations [54]; however, xCS PRO:XYL membranes (ca. 0.090 kg m^−2^ h^−1^) are competitive compared to other reported membranes based on hydrophilic polymers (CS, polyimide, PEEKWC). On the contrary, PLA, PVA and polyarylethersulfone with cardo filled with [Cu_2_(bdc)_2_(bpy)]_n_ still stand as more permeable for this organic–organic separation. To some extent, it seems that CS membranes blended with DESs present great potential for the extremely challenging organic–organic separation via pervaporation; however, more efforts must be focused on optimizing the membrane structure to overcome the limitation of low permeation while maintaining the selective properties.

## 4. Conclusions

In this work, we evaluated the potential of three different deep eutectic solvent (DES) formulations in fabricating dense CS membrane films. According to the microscopic characterization, all membrane formulations presented a dense structure, indicating that this pattern was not greatly modified by the DES incorporation, while their crystallinity and mechanical properties were substantially influenced. However, according to the surface analysis (contact angle), the main contribution of the DESs has been observed on the membrane surfaces, on which, depending on the DES precursors and their polarity, the film membranes exhibited different contact-angle measurements, interpreted as hydrophilic or hydrophobic. Additionally, the membranes also benefited from improved selective properties toward MeOH. In comparison with pristine CS, the membranes doped with DES showed an improved separation factor (which was even further improved by applying the crosslinking) and permeation rates. To some extent, the permeate flux and the separation factor were dependent on the feed temperature as well. In conclusion, this work confirms the potentiality of improving CS membrane performance by applying green additives, like DES, which were fully compatible with the investigated biopolymer chitosan.

## Figures and Tables

**Table 1:** Characteristics of the investigated DES.

DES Composition			DES Chemical Structure		DES Code
HBA	HBD	Molar Ratio (HBD:HBA)	HBA	HBD	
Proline	Glucose	5:1	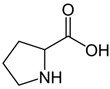	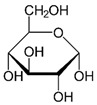	PRO:GLU
2-pyrrolidone-5-carboxylic acid	Sulfolane	1:3	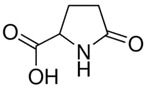	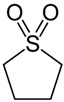	PCA:SULF
Proline	Xylitol	5:1	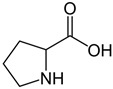	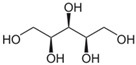	PRO:XYL

**Table 2:** Apparent activation energies for total flux, MeOH and MTBE partial fluxes.

Membrane	Activation Energy (E_a_) Values (KJ/mol)		
	Total	MeOH	MTBE
CS	7.89	5.10	20.02
xCS	12.20	13.07	15.33
CS PRO:GLU	3.23	1.33	7.63
xCS PRO:GLU	4.53	3.64	7.96
CS PCA:SULF	3.02	2.35	5.99
xCS PCA:SULF	3.18	3.23	7.03
CS PRO:XYL	4.65	4.43	5.42
xCS PRO:XYL	6.51	5.42	11.84

**Table 3:** PV membranes used for the selective separation of MeOH-MTBE.

Membrane Type	Filler Loading	MeOH Concentration	Operating Conditions:	J (kg m^−2^ h^−1^)	Separation Factor (α)	Ref.
GO-polyimide	4 wt%	4.3 wt% MeOH	45 °C, 0.05 mbar	0.091	9.0	[55]
PEEKWC	-	15 wt% MeOH	40 °C, 6.1 mbar	0.068	10	[56]
PVA	-	30 wt% MeOH	45 °C, 15 mbar	0.900	25	[57]
PLA	-	15 wt% MeOH	30 °C, 6 mbar	0.620	5	[51]
xCS:PRO:SUF	-	14.3 wt% MeOH	25 °C, 0.05 mbar	0.008	35.4	[8]
CS	-	30 wt% MeOH	50 °C	0.120	7	[58]
Sulfonated polyarylethersulfone with cardo filled with [Cu_2_(bdc)_2_(bpy)]_n_	30 wt%	15 wt% MeOH	40 °C, 6 mbar	0.28	2300	[59]
xCS PRO:XYL	-	14.3 wt% MeOH	25 °C, 0.05 mbar	0.090	36.4	This work

## Data Availability

The data presented in this study are available on request from the corresponding author.

## Supplementary Materials

The following supporting information can be downloaded at: https://www.mdpi.com/article/10.3390/membranes14110237/s1, Figure S1: FT-IR spectrum of CS membrane; Figure S2. FT-IR spectrum of xCS membrane; Figure S3. FT-IR spectrum of CS PRO:GLU membrane; Figure S4. FT-IR spectrum of xCS PRO:GLU membrane; Figure S5. FT-IR spectrum of CS PCA:SULF membrane; Figure S6. FT-IR spectrum of xCS PCA:SULF membrane; Figure S7. FT-IR spectrum of CS PRO:XYL membrane; Figure S8. FT-IR spectrum of xCS PRO:XYL membrane.
