# Supplementary material for: Enhancing the Separation Performance of Chitosan Membranes Through the Blending with Deep Eutectic Solvents for the Pervaporation of Polar/Non-Polar Organic Mixtures
Source: Membranes (Basel). 2024 Nov 11;14(11):237. doi: 10.3390/membranes14110237 (PMC11596667; doi:10.3390/membranes14110237)
Supplement: Supplementary file 1 [file membranes-14-00237-s001.zip › membranes-3304386-supplementary.pdf]

Supporting information

Enhancing the separation performance of chitosan membranes through the blending with deep eutectic solvents for the pervaporation of polar/non-polar organic mixtures

Francesco Galiano[1*], Asma Msahel[2], Francesca Russo[1], Natalia Rovella[1,3], Alfonso Policicchio[4], Sofiane Ben Hamouda[5], Amor Hafiane[2], Roberto Castro-Muñoz[6*], Alberto Figoli[1]

[1] Institute on Membrane Technology (CNR-ITM), Via P. Bucci 17/c, 87036 Rende, CS, Italy

[2] Laboratory of Water Membrane and Environmental Biotechnology (LMBE), CERTE BP 273, 8020 Soliman, Tunisia

[3] Department of Biology, Ecology and Earth Sciences (DiBEST), University of Calabria, Via P. Bucci 12/b, 87036 Rende, CS, Italy

[4] Department of Physics, Università della Calabria, Via P. Bucci, Cubo 31C, 87036, Rende (CS), Italy

[5] Centre for Research on Microelectronics and Nanotechnology (CRMN) of Sousse, 4054, Sousse, Tunisia

[6] Faculty of Civil and Environmental Engineering, Department of Sanitary Engineering, Gdansk University of Technology, 11/12 Narutowicza St., 80-233, Gdansk, Poland

Corresponding author: f.galiano@itm.cnr.it; food.biotechnology88@gmail.com;

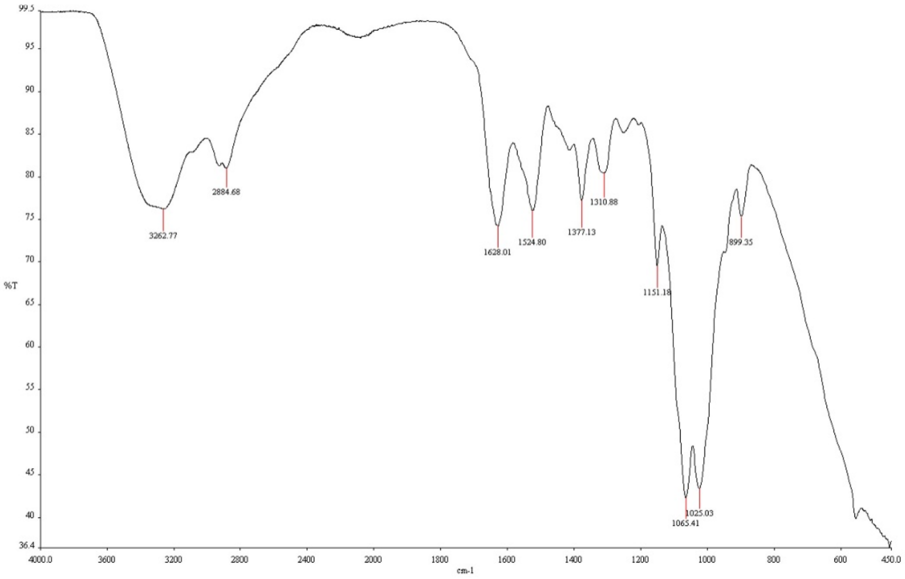

Figure S1. FT-IR spectrum of CS membrane

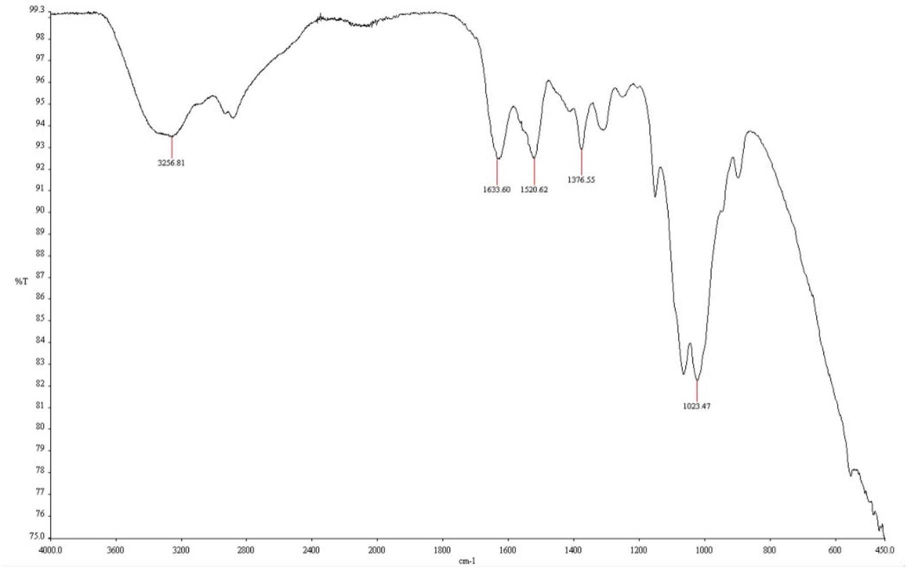

Figure S2. FT-IR spectrum of xCS membrane

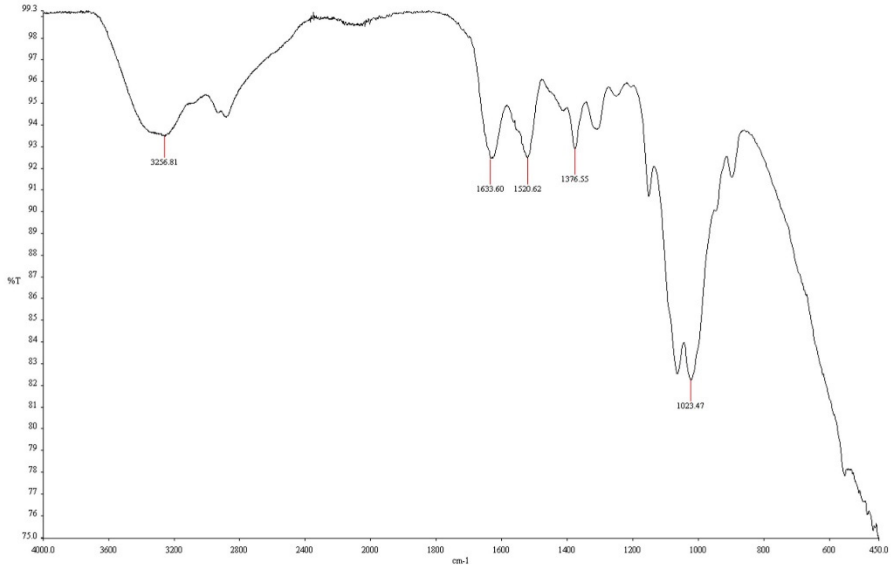

Figure S3. FT-IR spectrum of CS PRO:GLU membrane

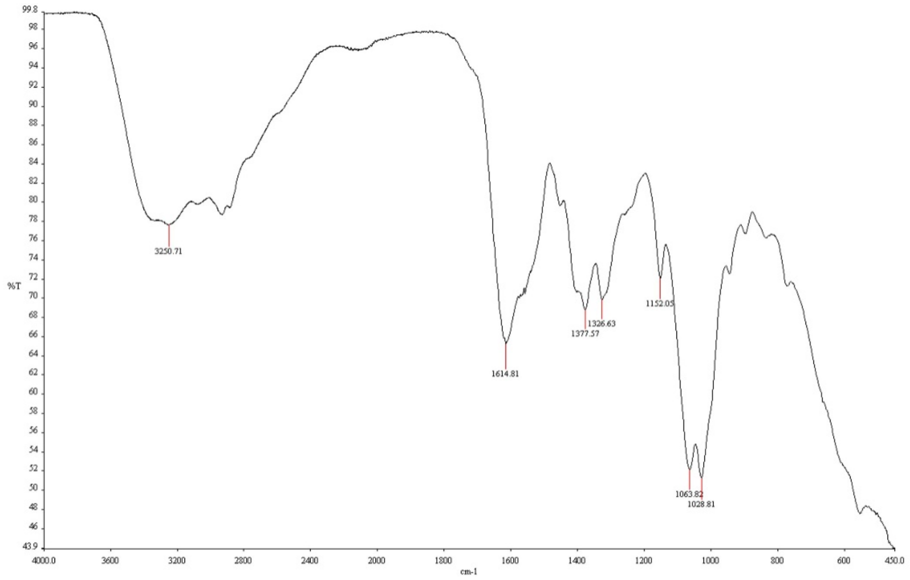

Figure S4. FT-IR spectrum of xCS PRO:GLU membrane

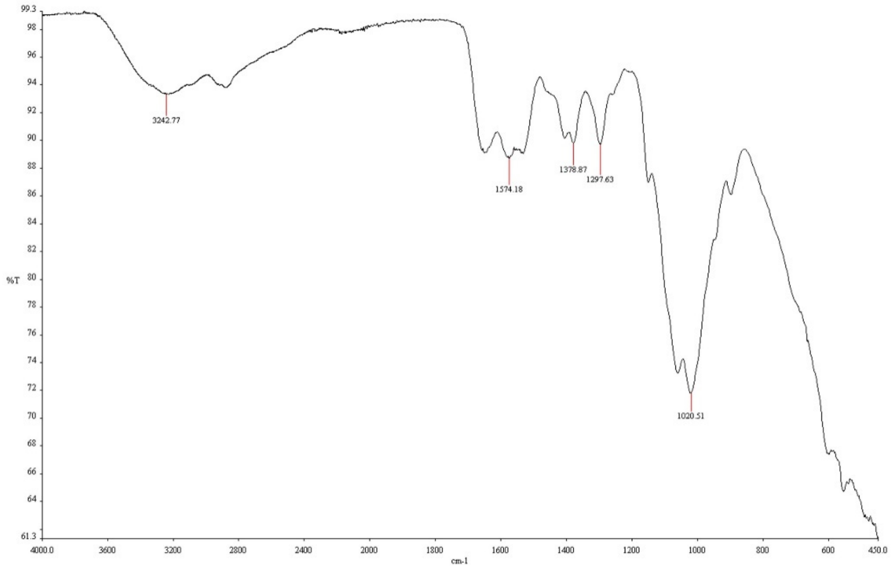

Figure S5. FT-IR spectrum of CS PCA:SULF membrane

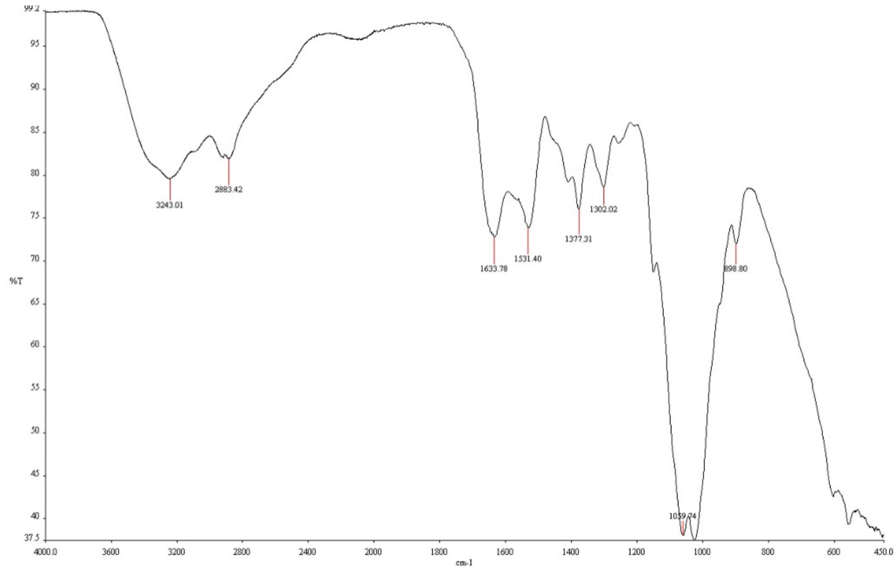

Figure S6. FT-IR spectrum of xCS PCA:SULF membrane

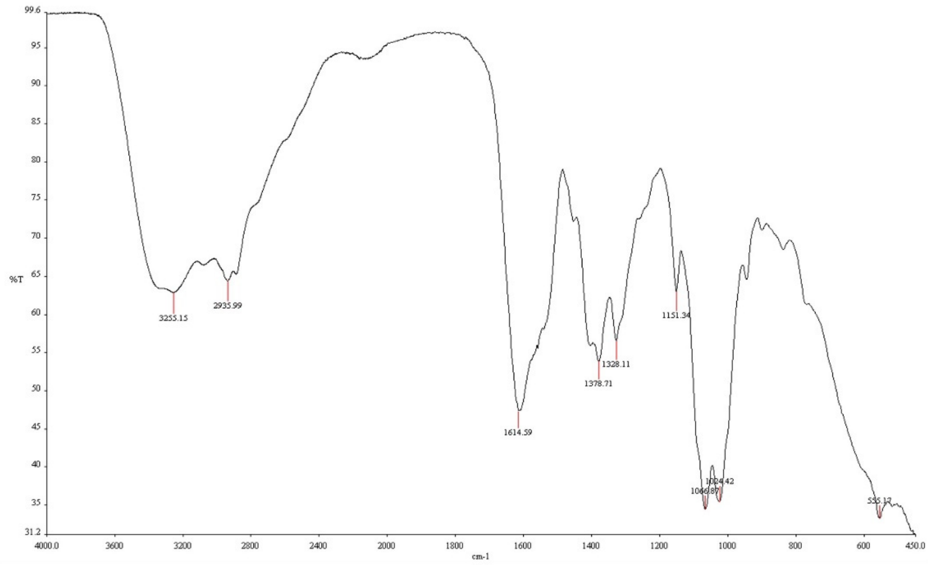

Figure S7. FT-IR spectrum of CS PRO:XYL membrane

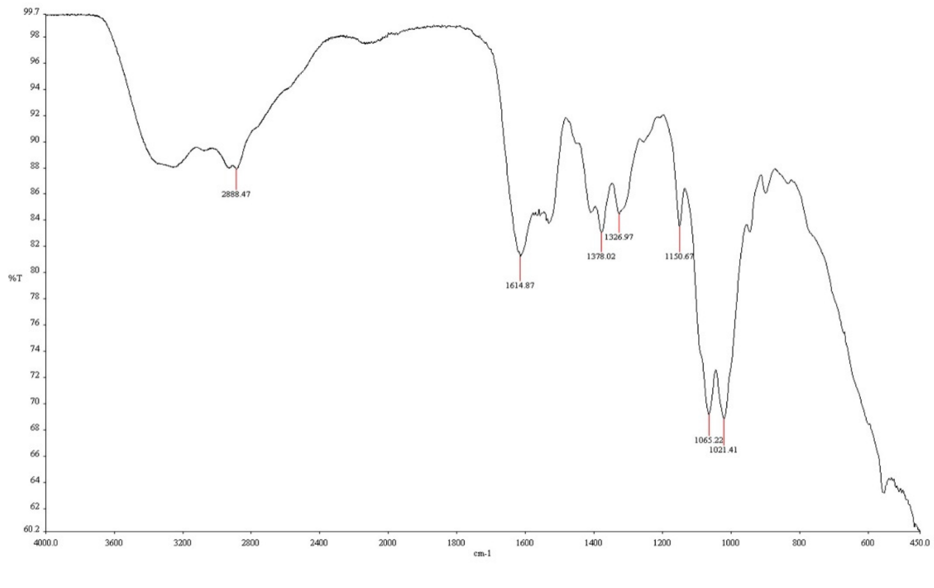

Figure S8. FT-IR spectrum of xCS PRO:XYL membrane
